# Low-Patching-Error Fiber Bragg Grating Multi-Dimensional Force Sensor

**DOI:** 10.3390/s26154908

**Published:** 2026-08-04

**Authors:** Shu Jiang, Huachuan Huang, Tao Li, Zhibin Ye, Shiyu Yang, Deming Hu

**Affiliations:** 1 College of Electrical and Information Engineering, Quzhou University, Quzhou 324000, China; retcheywcs@aliyun.com (S.J.);; 2 Key Laboratory of Testing Technology for Manufacturing Process, Ministry of Education, School of Manufacturing Science and Engineering, Southwest University of Science and Technology, Mianyang 621010, China; huanghc@swust.edu.cn (H.H.);; 3 Shanghai Marine Equipment Research Institute, Shanghai 200031, China; litao117@sina.cn

**Keywords:** fiber Bragg grating, multi-dimensional force sensor, optimization of elastic element structure, crossbeam, patch error

## Abstract

In order to avoid the difficulties caused by patching-errors in fiber Bragg grating multi-dimensional force sensors, this paper applies equal strength beam structure to a crossbeam elastic element, designs and implements an improved fiber Bragg grating multi-dimensional force sensor and constructs a calibration testing system to load and test the sensor. The results show that the sensitivity change rate of the improved elastic body is significantly better than that of the traditional crossbeam elastic element. The sensitivity change rate in the X or Y directions is only about 11% of the traditional crossbeam, and the z-direction is only about 35% of that of the traditional crossbeam, greatly reducing the requirement for the accuracy of sensitive component patching. The repeatability and the hysteresis of the FBG three-dimensional force sensor are better than 1% of the full scale (200 N). The measurement error of the improved sensor in the X direction is 0.27 N, which is lower than the 0.9 N of the traditional crossbeam sensor. In the Z direction, the measurement error of the improved sensor is 0.62 N, significantly lower than the 5.73 N of the traditional crossbeam. In summary, the measurement accuracy of the FBG three-dimensional force sensor based on the improved elastic element has been significantly improved. The work presented in this article can also be applied to other forms of multi-dimensional force sensors, providing important technical references for the mass production and performance improvement of multi-dimensional force sensors, and has significant engineering implications.

## 1. Introduction

In recent years, multi-dimensional force sensors have been widely applied in the field of robotics. A robot can achieve more precise and complex operations by installing multi-dimensional force sensors on its wrist, which provide real-time feedback on force perception information in the environment to the robot control system [1,2]. Robots often work in complex and uncertain environments, making it difficult to obtain environmental information. Developing multi-dimensional force sensors that work stably and reliably in various complex environments is one of the important challenges in developing intelligent robots [3].

### 1.1. Measurement Principle of Multi-Dimensional Force Sensor

According to the measurement principle, multi-dimensional force sensors can be divided into a resistance type [4], piezoelectric type [5], capacitance type [6], optical fiber type [7], etc.

A resistance strain gauge is one of the most widely used in the field at present. It has a series of advantages, such as high precision, a wide measurement range, mature technology, etc., but it also has some disadvantages, such as a poor dynamic response, the bridge circuit is greatly affected by external factors, etc.

A piezoelectric multi-dimensional force sensor is a potential sensor that uses the principle of piezoelectric effect to measure the force/torque. It is one of the most promising sensors in the development of intelligent structure and robot technology [8]. The piezoelectric effect is usually used in the manufacture of tactile sensors [9,10]. At present, Xu has carried out research on using piezoelectric elements as sensitive elements to sense the measured force/torque [11,12,13]. The main characteristic of piezoelectric material is that it has a very high natural frequency, which is especially suitable for dynamic measurement, but its largest disadvantage is that it cannot act on static force for a long time [14].

The capacitive multi-dimensional force sensor measures the multi-dimensional force by setting a pair of capacitors and changing the relative gap between the electrode sheets [15]. Its advantage is that it has high sensitivity and flexibility, but because it cannot get rid of the problems of poor anti-interference and parasitic capacitance, such sensors are relatively less used in practical applications.

As an important member of optical fiber sensing, fiber Bragg grating (FBG) is widely used in large-scale infrastructure projects [16,17], aerospace [18], shipbuilding and marine engineering [19] and other fields. It has the advantages of no electricity detection, no electromagnetic interference, high and low temperature resistance, no zero drift, high precision, small volume, extremely stable physical properties, good biocompatibility, and good disinfection performance; one fiber can be connected with multiple grating measuring points in series to realize distributed measurement, and wavelength coding is adopted without being affected by the power fluctuation of signal light source. These advantages meet the requirements of robots for measuring elements and have quickly become a research hotspot [20,21,22,23,24,25,26,27].

### 1.2. Elastic Element Structure of Multi-Dimensional Force Sensor

In the research on multi-dimensional force sensors, the design of the elastic element structure is the core problem, including the vertical beam type, crossbeam type, cylinder type and so on.

The crossbeam elastic element takes into account both the horizontal and vertical strain effects and is a typical structure of the elastic element of the multi-dimensional force sensor [28,29]. Its advantages are high symmetry, a compact structure, high stiffness, and easy processing, while its disadvantages are dimensional coupling and the radial effect.

The spoke-type elastic element has the advantage of high stiffness, but its output exhibits significant nonlinearity [30].

The cylindrical elastic element has small dimensional coupling, but its stiffness is low [31].

Some scholars have made improvements on these basic structures, such as processing dumbbell shaped grooves on the crossbeam elastic element [32] and developed a large range six-dimensional force sensor to ensure high sensitivity while ensuring a large range. Hexiaohui et al. removed the floating beam of the traditional crossbeam six-dimensional force sensor and designed a six-dimensional force sensor with a sliding structure elastic element in combination with the characteristics of measuring a small force with a two-hole parallel beam and eliminating crosstalk by sliding [33].

### 1.3. Existing Problems

Specifically, the existing multi-dimensional force sensors have the following problems.

(1) Multi-dimensional force sensor elastic element performance is difficult to improve.

The performance of the elastic element is one of the most important factors that directly affects the performance parameters of a multi-dimensional force sensor; so, its design is very important. From the perspective of the control system, the higher the accuracy of the detection and sensing system, the better the dynamic response, and the wider the measurement range, but from the perspective of the realization of the sensing system, there are contradictions between the performance parameters, and it is difficult to meet these requirements in practical application.

Due to the uneven strain distribution on the surface of the sensor elastic element, the patching error of the component can exacerbate the inter-dimensional coupling, significantly affecting the detection results [34,35]. Since the FBG component is larger in size than the strain gauge, this effect is even more pronounced. Although measurement errors can be controlled through algorithmic correction [36] or accurate attachment operations [37,38], they do not eliminate the source of error at its root and are not conducive to the mass production of sensors [39].

(2) The coupling mechanism between dimensions is complex, and modeling is difficult to eliminate.

Dimensional coupling is the main factor affecting the accuracy of a multi-dimensional force sensor. The dimensional coupling of complex force sensing system also shows the characteristics of nonlinearity. If this nonlinear effect is not eliminated or compensated, it will affect the performance of the sensor and even cause the misoperation of the robot control system.

However, the coupling mechanism between dimensions of most multi-dimensional force sensing systems is very complex, and it is difficult to analyze and model with theoretical analysis and system identification methods. At present, the most commonly used method of decoupling between dimensions is the linear decoupling method, which can greatly reduce the coupling between dimensions, but it is difficult to completely eliminate it. Some scholars have also proposed a nonlinear decoupling method, but these methods have a complex process and a large amount of calculation, which makes it difficult to meet the requirements of dynamic measurement.

### 1.4. Main Work of This Paper

Aiming at the problem of the patching error, based on the widely used and researched crossbeam elastic element, this paper proposes a multi-dimensional force sensor elastic element structure optimization method, reveals the law that the strain distribution of the sensor elastic element changes with the elastic element parameters, and designs an improved elastic element structure with low sensitivity change rate. The FBG three-dimensional force sensor is realized by using fiber Bragg grating as the sensitive element, and the calibration test system is constructed to test the sensor. The results indicate that the sensitivity change rate of the improved elastic element in the core region is significantly lower than that of the traditional crossbeam, and it has higher linearity. This technology can also be applied to the elastic element optimization of various beam type multi-dimensional force sensors; so, the work of this paper provides strong technical support for the engineering application of FBG multi-dimensional force sensors.

## 2. Design

The elastic element structure is the difficulty and core of multi-dimensional force sensor design. Based on the traditional crossbeam elastic element, this paper designs a low-patching-error FBG three-dimensional force sensor.

### 2.1. FBG Three-Dimensional Force Measurement Principle

**Figure 1:**
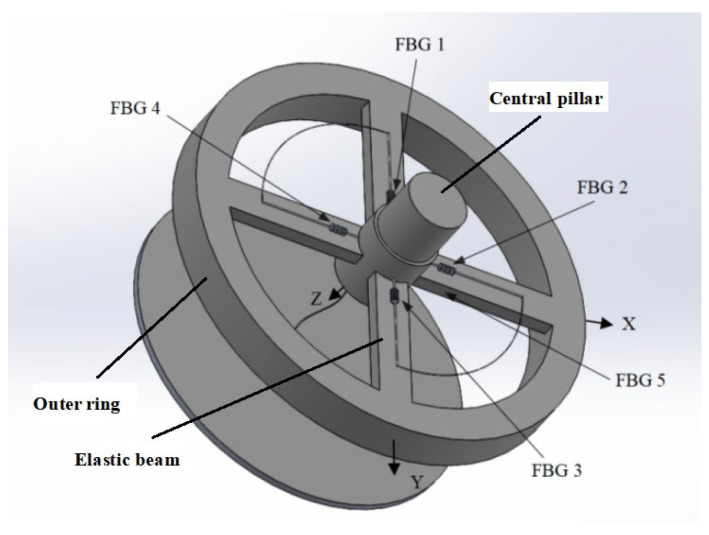
Schematic diagram of FBG three-dimensional force sensor.

As shown in Figure 1, the elastic element consists of four rectangular beams, one central column, and one outer ring. The threaded part at one end of the central column is directly subjected to various external forces ($F_{x}$, $F_{y}$ and $F_{z}$), causing the four rectangular beams to produce a certain amount of strain.

According to the scheme shown in Figure 1, FBG sensing elements are arranged. Four sensing elements, FBG1,FBG2, FBG3, and FBG4, are placed on the upper surfaces of Beam 1, Beam 2, Beam 3, and Beam 4, respectively. The sensing element FBG5 is placed on the lower surface of Beam 2. Ignoring the influence of temperature changes, the change in the central wavelength of the FBG sensing element Δλ is directly proportional to the magnitude of the strain ε on the beam; that is, $\Delta \lambda =k_{\varepsilon }\varepsilon$, where $k_{\varepsilon }$ is the strain sensitivity of the fiber Bragg grating, typically around 1.2 pm/με.

When *F_x_* acts, FBG2 and FBG4 are located in two regions with opposite strain directions, resulting in wavelength shifts of the same magnitude but opposite direction; that is, $\Delta \lambda_{4}=k_{\varepsilon }\varepsilon$, and $\Delta \lambda_{2}=-k_{\varepsilon }\varepsilon$. $\Delta \lambda_{4}$ and $\Delta \lambda_{2}$ represent the wavelength changes of FBG4 and FBG2, respectively. When *F_y_* or *F_z_* is applied, these two regions either do not produce strain or produce exactly the same strain. The difference in wavelength changes between FBG4 and FBG2 is denoted as $\Delta \lambda_{4-2}$; that is,

(1) $$\Delta \lambda_{4-2}=2k_{\varepsilon }\varepsilon_{x}.$$

This difference in wavelength change is only sensitive to $F_{x}$ and can be used as a measurement signal for $F_{x}$.

Similarly, the difference in wavelength change $\Delta \lambda_{1-3}=\Delta \lambda_{1}-\Delta \lambda_{3}$ can be used as a measurement signal for $F_{y}$. Furthermore, the difference in wavelength change $\Delta \lambda_{4-5}=\Delta \lambda_{4}-\Delta \lambda_{5}$ is insensitive to both $F_{x}$ and $F_{y}$ but only sensitive to $F_{z}$, and thus, it can be used as a measurement signal for $F_{z}$. Therefore, we have

(2) $$\begin{array}{l} F_{x}=k_{x}\Delta \lambda_{4-2}\\ F_{y}=k_{y}\Delta \lambda_{1-3}\\ F_{z}=k_{z}\Delta \lambda_{4-5} \end{array}$$

In the formula, $k_{x}$, $k_{y}$ and $k_{z}$ represent the sensitivities of the multi-dimensional force sensor in the *F_x_*, *F_y_*, and *F_z_* directions, respectively, with the unit of N/pm.

### 2.2. Traditional Crossbeam Theoretical Model

Due to the symmetrical distribution of the crossbeam elastic element, the deformations caused by *F_x_* and *F_y_* are similar. Therefore, this article establishes a mechanical model of the sensor’s elastic beam deformation when subjected to *F_x_* and *F_z_* to analyze the changes in the sensor.

**Figure 2:**
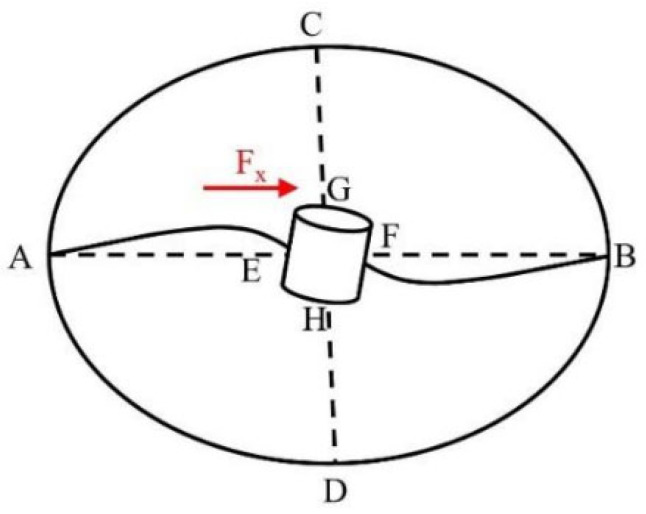
Schematic diagram of force on crossbeam in *F_x_* direction.

As shown in Figure 2, when the top of the central cylinder is subjected to a force (*F_x_*) in the X direction, it is equivalent to generating a moment *M* around the y-axis on the elastic beam. At the same time, beams AE and BF will be subjected to tension and compression in the x direction, respectively, which is equivalent to generating a bending moment around the y-axis on the sensor. In addition, beams CG and DH will also experience a torque rotating around the y-axis, but the resulting effect is negligible and can be ignored. among which

(3) $$M=F_{x}H.$$

In the formula, M represents the moment applied to the central cylinder, *F_x_* denotes the force in the X-direction applied to the top of the central cylinder, and H signifies the height of the central cylinder.

Due to the moment applied to the beam, both sides of the beam experience equal and opposite reaction forces, which can be expressed as follows:

(4) $$F_{A}=F_{B}=\frac{M}{2(l+r)}.$$

Using the section method, the bending moment on the section located at a distance *x* away from support A can be expressed as

(5) $$M(x)=F_{A}x.$$

Combining Formulas (3) and (4), and according to material mechanics, the surface strain of the beam at a position x away from support A or B can be expressed as [40]

(6) $$\varepsilon_{x}=\frac{\sigma }{E}=\frac{M(x)}{EW_{z}}=\frac{3F_{x}Hx}{Ebh^{2}(l+r)}.$$

In the formula, *ε_x_* represents the strain at a position on the elastic beam that is *x* away from the support, *W_z_* denotes the bending section modulus of the elastic beam, *F_x_* signifies the force applied to the top of the central cylinder in the x direction, E stands for the elastic modulus of the elastic beam, l indicates the length of the elastic beam, *b* signifies the width of the elastic beam, *h* represents the thickness of the elastic beam, and *r* represents the radius of the central cylinder.

Under the action of *F_x_*, the strain values on the surfaces of beam 2 and beam 4 are equal in magnitude but opposite in direction. The measured signal $\Delta \lambda_{4-2}$ corresponding to the strain value is $2\varepsilon_{x}$, and by combining Equations (1), (2), and (6), we can obtain

(7) $$k_{x}=k_{y}=\frac{Ebh^{2}(l+r)}{6Hxk_{\varepsilon }}.$$

**Figure 3:**
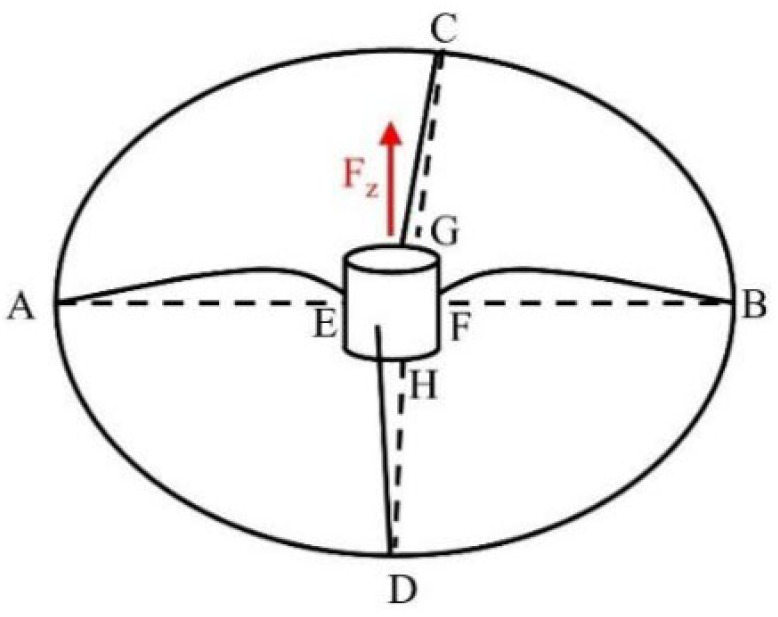
Schematic diagram of force on crossbeam in *F_z_* direction.

When the top of the central cylinder is subjected to an axial force in the *F_z_* direction, due to the centrosymmetric structure of the sensor’s elastic beams, the strain conditions of beams AE, BF, CG, and DH are all the same. Taking elastic beams AE and BF as examples for strain analysis, the load borne by the center of the central cylinder is *F* = *F_z_*/2. The simplified mechanical model (under the force *F_z_*) is shown in Figure 3:

The elastic beam only bears a concentrated load at the middle of the central cylinder. Therefore, the reaction forces on both sides of the beam are equal in direction and magnitude, which can be expressed as

(8) $$F_{A}=F_{B}=\frac{F}{2}=\frac{F_{z}}{4}.$$

From the section method, it can be known that the bending moment on the section located at a distance *x* away from support A or B can be expressed as

(9) $$M(x)=F_{A}x.$$

According to Formulas (6) and (7), and based on material mechanics, the strain on the beam surface at a position *x* away from support A or B can be expressed as

(10) $$\varepsilon_{z}=\frac{\sigma }{E}=\frac{M(x)}{EW_{z}}=\frac{3F_{z}x}{2Ebh^{2}}.$$

In the formula, *ε_z_* represents the strain at a position on the elastic beam that is *x* away from the support, *W_z_* denotes the bending section coefficient of the elastic beam, *F_z_* signifies the force in the Z direction applied on the top of the central cylinder, *x* indicates the distance from support A or B, E stands for the elastic modulus of the elastic beam, *b* represents the width of the elastic beam, and *h* is the thickness of the elastic beam.

Similarly to $k_{x}$ and $k_{y}$, the sensitivity of $F_{z}$ is

(11) $$k_{z}=\frac{Ebh^{2}}{3xk_{\varepsilon }}.$$

**Figure 4:**
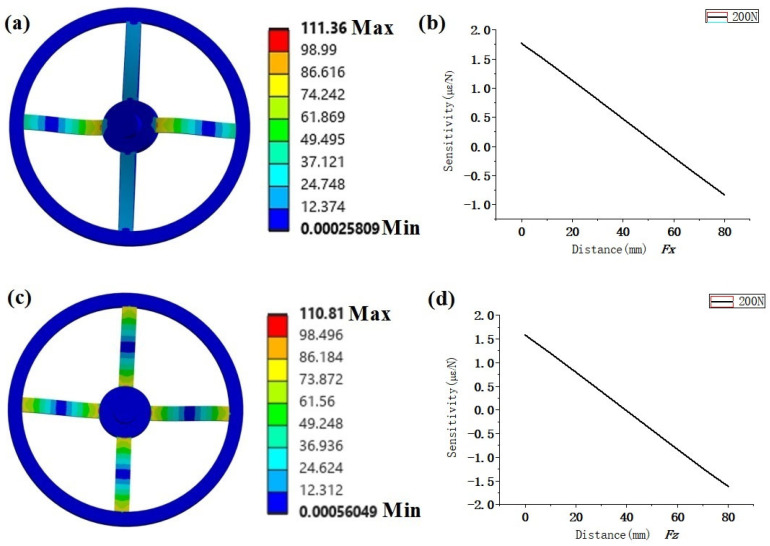
Traditional crossbeam statics simulation diagram. (**a**) Static simulation cloud image of traditional cross beam (*F_x_* direction). (**b**) Traditional cross beam strain sensitivity distribution (*F_x_* direction). (**c**) Static simulation cloud image of traditional cross beam (*F_z_* direction). (**d**) Traditional cross beam strain sensitivity distribution (*F_z_* direction).

According to the simulation results shown in Figure 4, the strain distribution from the central column to the outer ring exhibits a linear variation trend. The strain gradient on the beam is significant, and the bonding position of the sensitive element has a notable impact on the sensor sensitivity. The suitable bonding area on the beam is limited to a small section near the central column, posing difficulties for sensor packaging and manufacturing and limiting mass production.

### 2.3. Optimization Design of Elastic Element Structure

**Figure 5:**
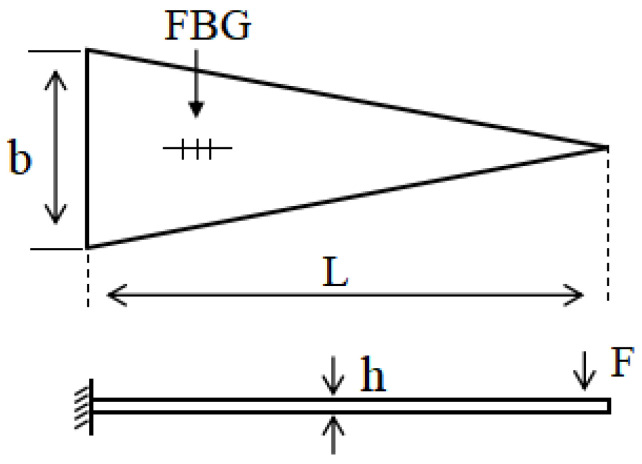
Schematic diagram of equal-strength cantilever beam.

An equal-strength cantilever beam exhibits a characteristic of uniform surface strain distribution when the free end vertex is subjected to force, as illustrated in Figure 5. Due to the force applied to the free end of the beam with equal strength, the axial strain at the surface test point is as follows:

(12) $$\varepsilon =\frac{6FL}{Ebh^{2}},$$

where *ε* represents the strain of the cantilever beam at the position where the optical fiber is pasted; *E* is related to the elastic modulus and material properties; *F* denotes the force applied to the free end of the cantilever beam; *b* stands for the width of the fixed end of the cantilever beam; *L* and *h* represent the height and thickness of the cantilever beam, respectively.

**Figure 6:**
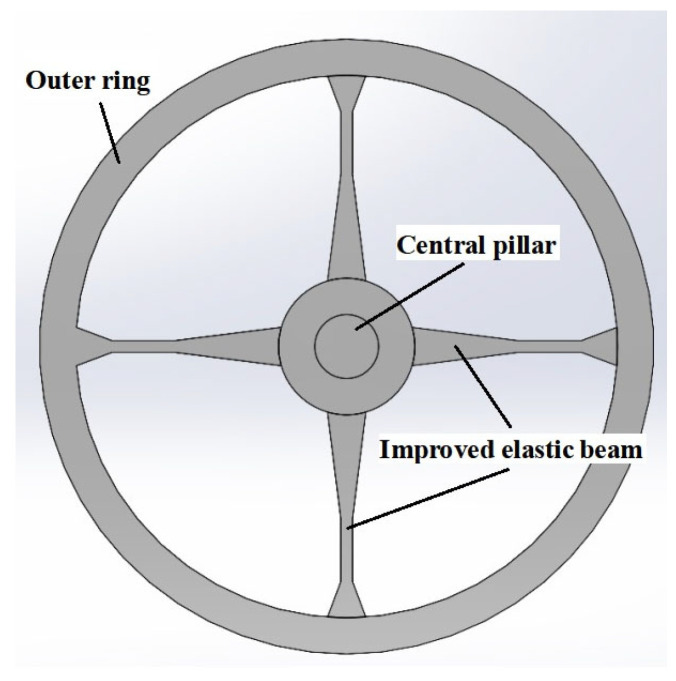
Improved crossbeam elastic element.

To achieve a more uniform strain distribution on the elastic element surface, expand the area suitable for patch attachment, and minimize the impact of patching errors on sensor performance, this paper applies the isosceles triangular structure of an equal-strength beam to the crossbeam elastic element, forming an improved elastic element structure as shown in Figure 6. Establishing an analytical model for this structure is relatively complex, so finite element statics simulation is used to assist in the optimization of the elastic element topology [3].

This article selects 2Cr13 stainless steel as the material for the preparation of the elastic element. Its elastic modulus E is 210 GPa, Poisson’s ratio ν is 0.3, tensile strength is ≥540 MPa, and yield strength is ≥345 MPa. It possesses excellent elastic deformation capability, precision machining performance, and corrosion resistance.

Firstly, an initial geometric model is constructed using UG (NX6), ignoring non-critical details such as threads and chamfers during the modeling process to simplify the simulation calculations. Subsequently, the geometric model is imported into ANSYS (2015) software for mesh generation: a coarse mesh is first generated using tetrahedral elements for the entire elastic element, followed by mesh refinement for the four elastic beams (the core areas for force transmission and deformation sensing).

To ensure the accuracy and reliability of the finite element simulation results and avoid deviations caused by mesh density, this paper conducts a mesh independence verification for the elastic element model. The verification method is as follows. Under the premise of maintaining the boundary conditions and loads (*F_x_* = 200 N, full scale) unchanged, seven points in the strain path are selected to analyze strain changes, and the mesh size is gradually adjusted for comparative analysis. The mesh sizes are set to 4 mm, 3 mm, 2 mm, 1.5 mm, 1 mm, and 0.8 mm, respectively. The strain values under each mesh size are recorded, and the change rate is calculated. When the mesh size is gradually refined from 4.0 mm to 1.0 mm, the strain change rate gradually decreases. Further refinement of the mesh size does not result in significant fluctuations in strain changes, but the calculation time increases significantly. Considering both the calculation accuracy and efficiency, this paper chooses 1 mm as the mesh size for the core area of the elastic beam.

We add constraints to the elastic element, applying a fully fixed constraint (with both displacement and rotation being 0) to the outer surface of the fixed disc on the outer ring, simulating the fixed state of the elastic element after actual installation. Secondly, since the deformation and strain results of the elastic element under the actions of *F_x_* and *F_y_* are equivalent, this paper only applies a unidirectional graded load to the *F_x_* and *F_z_* directions. The load acts on the center of the upper surface of the central cylinder, with a load range of 20 N–200 N and a graded increment of 20 N, applied sequentially and solved. At the same time, a strain path is set on the mid-axis of the elastic beam surface for subsequent analysis.

**Figure 7:**
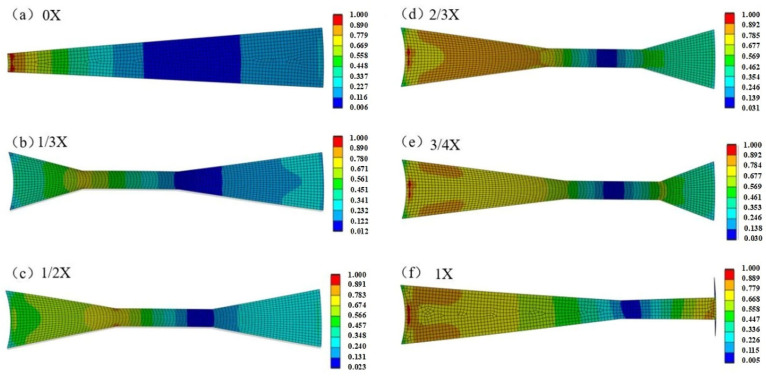
Improved verification of elastic element parameters for crossbeam (*F_x_*).

**Figure 8:**
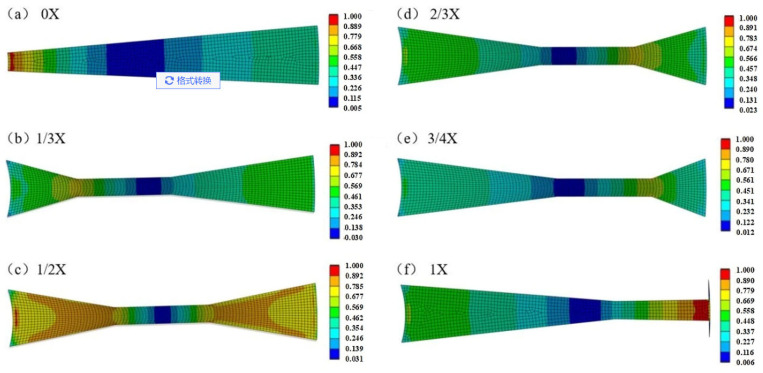
Improved verification of elastic element parameters for crossbeam (*F_z_*).

This article investigates the influence of the position of the apex of an isosceles triangle, the thickness of the beam, and the width of the beam root on the strain distribution pattern on the beam. Figure 7 and Figure 8 show the static simulation nephograms of strain distribution on the elastic body when the vertex is at positions 0*X*, 1/3*X*, 1/2*X*, 2/3*X*, 3/4*X*, and 1*X* (the total length of the beam is *X*) from the central column. Under the force in the x direction, as the vertex moves from the central column to the outer circle, the uniformity of strain gradually improves, reaching its best near the 3/4 position, and then deteriorates again. When subjected to force in the Z direction, the variation in the strain distribution on the beam with the position of the vertex also follows the same pattern.

To quantitatively compare the sensitivity distribution of elastic beams at different vertex positions, the uniformity of sensitivity distribution is quantitatively analyzed through the sensitivity change rate. The formula for calculating the sensitivity change rate is as follows:

(13) $$\eta_{s}=\frac{S_{\max}-S_{\min}}{\bar{S}};$$

(14) $$\bar{S}=\frac{\sum_{i=1}^{n}S_{i}}{n}.$$

The elastic element here is not equipped with sensitive components, and its sensitivity refers to the ratio of strain to force. In the formula, *η_s_* represents the sensitivity change rate (in %), *S_max_* and *S_min_* are the maximum and minimum sensitivities on the strain path, respectively, and $\bar{S}$ denotes the average sensitivity on the strain path (in με/N). $S_{i}$ is the strain sensitivity of the *i*th measurement point (out of *n*) on the strain path.

A standard strain path is set along the central axis of the beam body (from 0 mm to 80 mm from the central cylinder), and strain and strain data are extracted at each measuring point along the path at equal intervals. A key interval (with a total of 25 points) on the surface of the elastic beam, located between 20 mm and 40 mm from the central cylinder, is selected as the benchmark for sensitivity comparison. The sensitivity change rate within this interval is calculated using Formula (10) to quantitatively evaluate the uniformity of sensitivity distribution in beams with different vertex equal strengths. The results are shown in Table 1.

The sensitivity variation rate within the range of 20–40 mm clearly reflects the pattern mentioned above. The equal-strength beam with a 3/4*X* vertex position exhibits a sensitivity variation rate of only 0.95% in the *F_x_* direction and 32.76% in the *F_z_* direction within this core range, significantly outperforming other vertex configurations such as 0*X*, 1/3*X*, 1/2*X*, 2/3*X*, and 1*X*. Upon comprehensive comparison, the 3/4*X* vertex configuration emerges as an ideal solution for reducing patching errors.

**Figure 9:**
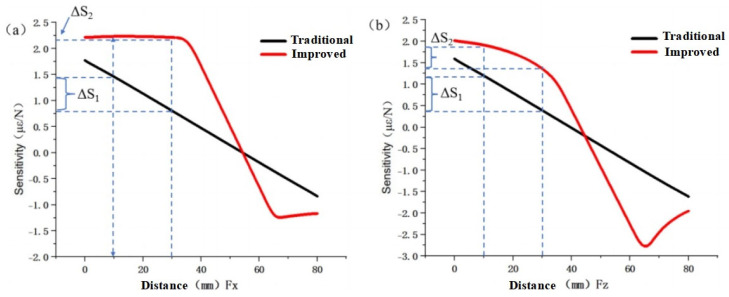
Comparison diagram of sensitivity distribution between traditional crossbeam and improved elastic element (3/4X). (**a**) *F_x_* direction. (**b**) *F_z_* direction.

The sensitivity distribution characteristics of a 3/4*X* vertex equal-strength beam compared to a traditional uniform-section beam are illustrated in Figure 9. Evidently, the traditional uniform-section beam exhibits a large sensitivity gradient and an extremely uneven distribution. Calculations reveal that within the range of 20–40 mm, the sensitivity change rate in the *F_x_* direction reaches 82.39%, and in the *F_z_* direction, it reaches 95.02%. In contrast, the 3/4*X* vertex equal strength beam achieved a significant reduction in sensitivity change rate within the core range through the structural design of an equal strength cross section. The sensitivity change rate in the *F_x_* direction is reduced by89.3% compared to the uniform-section beam, and in the *F_z_* direction, it is reduced by 65.5%. This structural approach addresses the core issue of uneven sensitivity distribution in traditional uniform-section beams.

**Figure 10:**
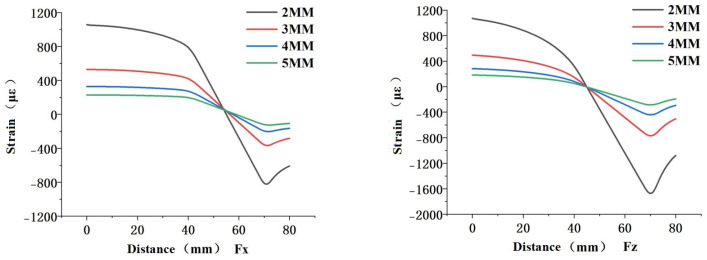
Strain distribution of beams with different thicknesses.

Figure 10 illustrates the strain distribution in a 3/4*X* vertex configuration across a beam thickness ranging from 2 mm to 5 mm. It is evident that the beam thickness only alters the magnitude of the deformation but does not affect the distribution pattern of strain on the beam.

**Figure 11:**
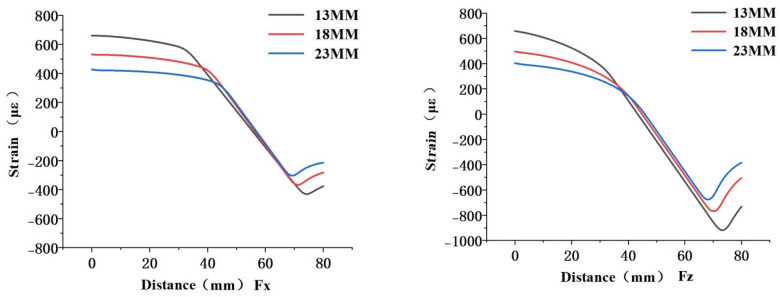
Strain distribution of beams with different widths.

Figure 11 illustrates the strain distribution in the 3/4*X* vertex configuration at the beam root with a width ranging from 13 mm to 23 mm. It can be seen that the beam width has little effect on the strain distribution pattern.

In summary, the width of the beam root and the thickness of the beam have insignificant effects on the strain distribution, while the position of the vertex of the isosceles triangle is the key factor affecting the uniformity of strain distribution on the beam.

The elastic element of the FBG three-dimensional force sensor designed in this paper features an outer diameter of 260 mm for the outer ring, four symmetrically distributed elastic beams with a length of *l* = 95 mm, a beam root width of 18 mm, and a beam thickness of 3 mm. The rated force values in the X, Y, and Z directions are 200 N.

### 2.4. FBG Packaging Design

**Figure 12:**
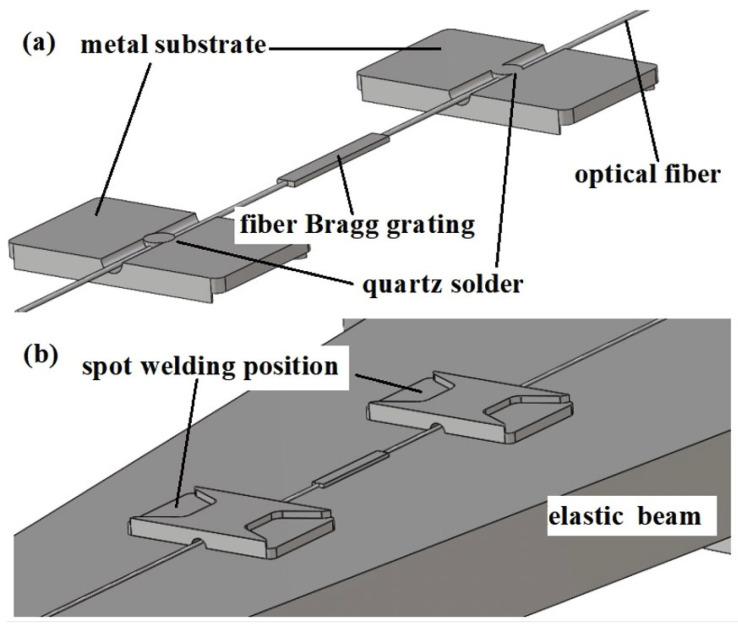
Schematic diagram of FBG sensor packaging. (**a**) FBG package component. (**b**) Installation diagram of packaging component on elastic beam.

The two ends of the fiber Bragg grating are encapsulated on two compact metal substrates, as shown in Figure 12, using low-temperature quartz solder. A fiber guiding groove is designed in the middle of the metal substrate, where the position for securing the fiber is the molten pool of quartz solder. The two sides of the substrate are designated for spot welding, with a thickness not exceeding 0.5 mm. The spot-welding positions on both sides align with the molten pool on a straight line, which is orthogonal to the guiding groove. After encapsulation, the metal substrate is spot welded to the designated position of the elastic beam. During the spot-welding process, the fiber Bragg grating is pre-stretched to facilitate the measurement of negative strain.

## 3. Verification

### 3.1. Design of a Three-Dimensional Force Sensor Calibration and Testing System

**Figure 13:**
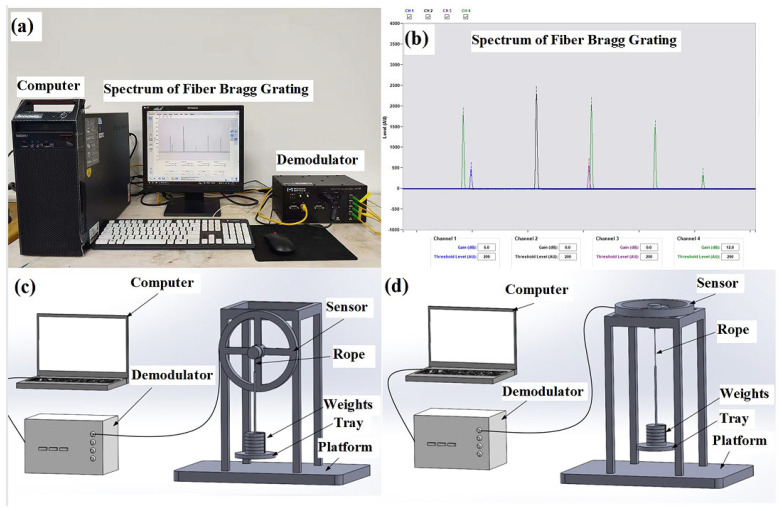
Static calibration experimental system for multi-dimensional force sensor. (**a**) Fiber Bragg grating demodulator and upper computer system. (**b**) Spectrum of Fiber Bragg Grating. (**c**) Three dimensional force sensor installed on the test system (X or Y direction). (**d**) Three dimensional force sensor installed on the test system (Z direction).

This article establishes a calibration experimental system, as shown in Figure 13, for the loading test of the FBG three-dimensional force sensor. The system mainly comprises a prototype of the FBG three-dimensional force sensor, a platform, weights, steel wire ropes, hanging plates, an FBG demodulator, and a host computer.

We fix the outer ring of the sensor on the vertical reference plane of the stand and hang a weight on the end surface of the central column to apply a force in the X (or Y) direction to the sensor. We fix the outer ring of the sensor on the horizontal reference plane of the stand and hang a weight on the end surface of the central column to apply a force in the Z direction to the sensor.

We apply a gravitational load using multiple standard weights (10 N), with an accuracy level of 0.1%. The FBG demodulator collects the wavelength signal from the sensor, which is displayed and recorded by the host computer. The demodulator used in this paper is SM-130 (MOI, Atlanta, GA, USA), with a wavelength range of 1510 nm~1590 nm, a resolution of 1 pm, and a stability of 2 pm.

### 3.2. Verification of Strain Distribution in Sensor Elastic Element

To verify the improvement effect of the improved crossbeam elastic element on the uniformity of sensitivity distribution, this paper selects three equidistant measuring points located at distances of 20 mm, 30 mm, and 40 mm from the end of the central cylinder along the length direction of the elastic beam for testing. As a comparative experiment, measurements are conducted on both the traditional crossbeam elastic element and the improved elastic element.

**Figure 14:**
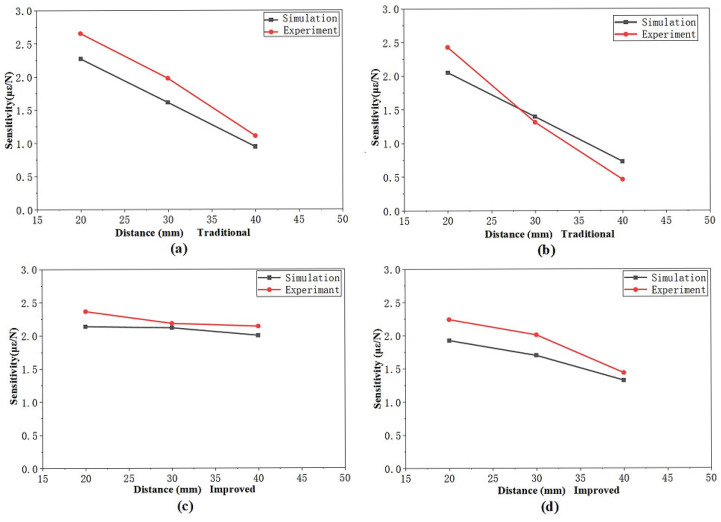
Comparison of measured and simulated strain sensitivity distribution of the two sensors. (**a**) Traditional cross beam sensor (X direction). (**b**) Traditional cross beam sensor (Z direction). (**c**) Improved sensor (X direction). (**d**) Improved sensor (Z direction).

The sensitivity of the three equidistant measuring points was calculated from experimental data, as shown in Figure 14. It can be observed that within the measurement range of 20 mm to 40 mm, the sensitivity of the improved elastic element changes slowly in both *F_x_* and *F_z_* directions. The further quantitative calculation of the sensitivity change rate of the three equidistant measuring points is shown in Table 2.

It can be clearly seen that the sensitivity change rate of the improved elastic element in the *F_x_* direction is only about 10% of that of the traditional crossbeam, and in the *F_z_* direction, it is only about 30% of that of the traditional crossbeam. This indicates that the strain values of the improved elastic element change gently within this range, with a significantly improved uniformity of distribution and small differences in sensitivity at various locations, strongly reducing the requirement for the precision of sensitive component bonding.

The experimental data and simulation data are highly consistent in terms of the magnitude and variation trend, both confirming that the improved elastic element exhibits significantly better strain uniformity on the beam compared to the traditional crossbeam. This design can significantly reduce the difficulty of patching during sensor packaging, essentially eliminating the adverse impact of patching errors on the accurate calculation of three-dimensional force values from a structural perspective, thus providing great convenience for the mass production of sensors.

### 3.3. Performance Testing of Improved Multi-Dimensional Force Sensor

The rated force values for the designed sensor in the X, Y, and Z directions are 200 N. The force value for each dimension starts at 20 N, increases by 20 N intervals to the rated value, and then gradually decreases by 20 N intervals to 0. This process is repeated at least three times, and the average value is taken as the measured value.

**Figure 15:**
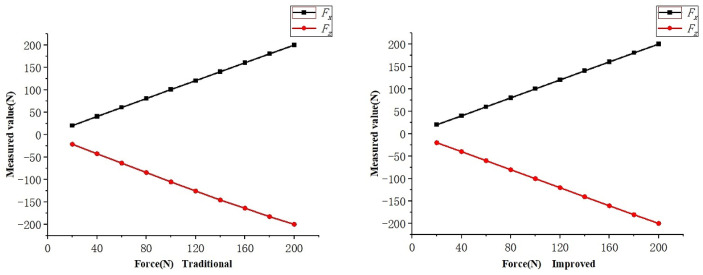
FBG three-dimensional force sensor loading test data.

We calculate the sensitivity of the FBG multi-dimensional force sensor according to Formula (1) and then obtain the measured values of each loading point. The experimental results are shown in Figure 15. The measurement error $I_{p}$, repeatability indicators $R_{F}$ and hysteresis $\nu_{H}$ are calculated according to the following formulas:

(15) $$I_{p}=F_{m}-F_{r};$$

(16) $$R_{F}=F_{max}-F_{min};$$

(17) $$\nu_{H}=\Delta F_{max}.$$

In the formula, $F_{m}$ represents the measured value of the three-dimensional force in N, $F_{r}$ represents the true value of the three-dimensional force in N, $F_{max}$ represents the maximum value of the three measured values in N, and $F_{min}$ represents the minimum value of the three measured values in N. $\Delta F_{max}$ is the maximum deviation between the measured values of forward and reverse travel in N.

The quantitative comparison of the measurement error and repeatability is shown in Table 3. It can be seen that the repeatability and the hysteresis of the FBG three-dimensional force sensor based on both elastic elements is better than 1% of the full scale, indicating that the elastic element material performance is qualified, and the experimental setup and experimental method are scientific and reasonable. The measurement error of the improved elastic element in the X direction is 0.27 N, which is lower than the 0.9 N of the traditional crossbeam, and in the Z direction, it is 0.62 N, much lower than the 5.73 N of the traditional crossbeam. In summary, it can be seen that the measurement accuracy of the FBG three-dimensional force sensor based on the improved elastic element has been significantly improved.

## 4. Discussion and Conclusions

Based on an improved crossbeam elastic element structure, this paper designs a novel FBG three-dimensional force sensor that achieves a low patching error. The performance parameters of the sensor are shown in Table 3. The sensor has a diameter of Φ260 mm and an axial dimension of 51 mm.

Due to its inherent characteristics, the electrical multi-dimensional force sensor is prone to electromagnetic interference, has poor insulation safety, and is not conducive to networking. As a passive optical device, fiber Bragg grating (FBG) has the advantages of intrinsic safety, immunity to electromagnetic interference, and convenient networking, gradually becoming an ideal technical approach for multi-dimensional force perception in robots.

However, multi-dimensional force sensors still suffer from the issue of patching errors, which arise from uneven strain distribution on the surface of the sensor’s elastic element. This significantly impacts the detection results. Some studies have attempted to reduce the measurement errors through algorithmic correction and improving the patching accuracy. Neither of these methods eliminates the source of errors, and the requirement to enhance the patching accuracy poses challenges for the mass production of sensors.

This article applies the equal strength beam structure to the traditional crossbeam elastic element and studies the strain distribution law on the elastic beam with the variation of elastic beam parameters through finite element static simulation. An improved elastic element with a significantly lower sensitivity change rate than the traditional crossbeam is designed. In the core range of the elastic beam (20 mm~40 mm), the sensitivity change rate of the improved elastic element in the *F_x_* direction is only 10% of that of the traditional crossbeam, and in the *F_z_* direction, it is 30%. This structure significantly reduces the requirement for the accuracy of sensitive component patching operation and can basically eliminate the measurement errors caused by surface patching position deviation.

The good measurement performance of the improved sensor has also been verified, such as good linearity, repeatability, and hysteresis. The repeatability and the hysteresis of the improved sensor are better than 1% of the full scale. The measurement error of the improved sensor in the X direction is 0.27 N, which is lower than the 0.9 N of the traditional sensor, and in the Z direction, it is 0.62 N, much lower than the 5.73 N of the traditional sensor. These results indicate that the measurement accuracy of the improved sensor has been improved compared to traditional sensors.

To facilitate operation, the FBG three-dimensional force sensor designed in this paper has a relatively large size. This issue can be addressed by adjusting the dimensions of the sensor components, such as the thickness and length of the beam. Furthermore, by using FBG sensing elements with shorter grating lengths (the grating length of the FBG used in this paper is approximately 10 mm), the sensor size can be further miniaturized. Therefore, if necessary, the size of the designed sensor can be significantly reduced.

Future work will encompass optimizing the sensor performance, reducing the sensor size, designing specialized connector extraction schemes, and utilizing sealing and waterproofing technologies to enhance the practicality of the sensors. Additionally, specialized fixtures will be developed to assist in the pre-tensioning of fiber Bragg gratings, thereby standardizing the packaging and manufacturing process of sensors. The technology presented in this paper can also be applied to improve elastic elements for other forms of multi-dimensional force sensors, demonstrating considerable practical value.

## 5. Patents

Chinese utility model patent: An improved sensor elastic element, ZL 2023 2 3190162.X.

## Figures and Tables

**Table 1:** Sensitivity change rate of different configurations (20–40 mm).

Configurations	Sensitivity Change Rate (*F_x_*)	Sensitivity Change Rate (*F_z_*)
Traditional	82.39%	95.02%
0X	104.81%	174.16%
1/3X	57.03%	105.00%
1/2X	20.17%	35.01%
2/3X	11.55%	36.11%
3/4X	8.78%	32.76%
1X	18.27%	34.93%

**Table 2:** Comparison of sensitivity change rate between improved elastic element and traditional crossbeam.

Direction	*F_x_*		*F_z_*	
Elastic Element Configuration	Traditional	Improved	Traditional	Improved
simulation	82.39%	8.78%	95.02%	32.76%
experiment	78.33%	9.73%	135.92%	43.35%

**Table 3:** Comparison of performance indicators of FBG three-dimensional force sensors.

Direction	*F_x_*		*F_z_*	
Sensor	Traditional	Improved	Traditional	Improved
Error (N)	0.9	0.27	5.73	0.62
Repeatability (N)	0.98	0.90	0.96	0.69
Hysteresis (N)	0.92	1.35	1.76	1.09

## Data Availability

The original contributions presented in this study are included in the article. Further inquiries can be directed to the corresponding author.

## Abbreviation

The following abbreviation is used in this manuscript:

FBG	fiber Bragg grating
